# C-Kit Cardiac Progenitor Cell Based Cell Sheet Improves Vascularization and Attenuates Cardiac Remodeling following Myocardial Infarction in Rats

**DOI:** 10.1155/2018/3536854

**Published:** 2018-06-25

**Authors:** K. Dergilev, Z. Tsokolaeva, P. Makarevich, I. Beloglazova, E. Zubkova, M. Boldyreva, E. Ratner, D. Dyikanov, M. Menshikov, A. Ovchinnikov, F. Ageev, Ye. Parfyonova

**Affiliations:** ^1^Laboratory of Angiogenesis, National Medical Research Center of Cardiology, Moscow, Russia; ^2^Laboratory of Gene and Cell Therapy, Institute of Regenerative Medicine, Lomonosov Moscow State University, Moscow, Russia; ^3^Laboratory of Gene and Cell Technology, Faculty of Medicine, Lomonosov Moscow State University, Moscow, Russia; ^4^Consultative and Diagnostic Department, National Medical Research Center of Cardiology, Moscow, Russia

## Abstract

The adult heart contains small populations of multipotent cardiac progenitor cells (CPC) that present a convenient and efficient resource for treatment of myocardial infarction. Several clinical studies of direct CPC delivery by injection have already been performed but showed low engraftment rate that limited beneficial effects of procedure. *«*Cell sheet*»* technology has been developed to facilitate longer retention of grafted cells and show new directions for cell-based therapy using this strategy. In this study we hypothesized that СPC-based cell sheet transplantation could improve regeneration after myocardial infarction. We demonstrated that c-kit+ CPC were able to form cell sheets on temperature-responsive surfaces. Cell sheet represented a well-organized structure, in which CPC survived, retained ability to proliferate, expressed progenitor cell marker Gata-4 formed connexin-43+ gap junctions, and were surrounded by significant amount of extracellular matrix proteins. Transplantation of cell sheets after myocardial infarction resulted in CPC engraftment as well as their proliferation, migration, and differentiation; cell sheets also stimulated neovascularization and cardiomyocyte proliferation in underlining myocardium and ameliorated left ventricular remodeling. Obtained data strongly supported potential use of CPC sheet transplantation for repair of damaged heart.

## 1. Introduction

Despite advances in treatment of chronic heart failure (CHF), it still remains severe and widely spread complications of cardiovascular disorders. Approximately 2% of the world's population suffers from CHF, yet this proportion increases annually. This observation encouraged researches to find new methods to reverse, repair, and revascularize failing heart tissue.

Transplantation of stem cells has emerged as a potential strategy to ameliorate ventricular remodeling and left ventricle dysfunction. Among many types of stem cells being investigated, c-kit+ resident CPC are considered as a promising candidate to regenerate damage heart. CPC that normally reside in myocardium are responsible for physiological cardiac cell turnover and able to differentiate into three main cardiac cell types (endothelial, smooth muscle cells, and cardiomyocytes)* in vitro* and* in vivo*. CPC transplantation improves cardiac function and reverses negative remodeling [1]. Completed SCIPIO trial (Stem Cell Infusion in Patients with Ischemic Cardiomyopathy) has demonstrated safety and promising potential of CPC for treatment of CHF [2].

Although preclinical studies and clinical trials have demonstrate potential of CPC to alleviate CHF, full potential of these cells has not been fully realized because of poor engraftment and survival. Local (intramyocardial or intracoronary injection) or systemic delivery may trigger loss of a considerable amount of the cells (up to 90%) due to either mechanical damage in the needle or apoptosis when the cells are administered into ischemic and inflamed tissues. In due course, accumulation of evidence led to reconsideration of mechanisms that determine the effects of cell therapy: currently cells are largely considered as a source of active biomolecules that trigger endogenous signaling pathways that control myocardial regeneration [3, 4]. This paracrine mechanism may render numerous effects (angiogenesis stimulation, reduction of fibrosis, inflammation and apoptosis, and recruitment of resident stem/progenitor cells) contributing to myocardial regeneration and restoration of contractile function. It should be mentioned that number of engrafted cells capable of performing their specific function is a crucial factor defining efficacy of therapy. Regenerative medicine has suggested application of *«*cell sheets*»* for delivery to increase cell survival after transplantation. Such *«*cell sheets*»* are mono- or multilayer tissue-engineered structures consisting of one or several types of cells and their extracellular matrix. It has been shown that stem/progenitor cell sheet transplantation provides higher efficiency and safety compared to multiple injections [5]. This method circumvents the limitations concerning the volume of injection, which promotes delivery of a greater number of cells to the area that requires therapeutic intervention. Another advantage of cell sheets is that the surface receptors tend to be preserved allowing more effective adhesion of transplanted cells to damaged tissue. In addition, cell sheets allow generating constructs that mimic specific tissue architectonics and cell-to-cell interactions* in vitro,* which improves cell survival and their engraftment to myocardium.

In this study we evaluated cell sheets as a method to improve survival and function of progenitor cells after transplantation and studied beneficial effects of c-kit+ CPC delivery in a rat model of myocardial infarction.

## 2. Methods 

### 2.1. Ethic Statement and Animal Strain Used

Wistar male rats (250-300 g) were purchased from “Puschino” SPF-grade facility (Puschino, Russia). Animals received food and water ratios according to in-house rules. Euthanasia was conducted by cervical dislocation after isoflurane narcotization. Manipulations were in compliance with EU Directive 2010/63/EU for animal experiments and approved by institutional ethics board (National Medical Research Center of Cardiology; permit #385.06.2009).

### 2.2. Isolation and Culture of c-Kit+ CPC from Rat Myocardium Samples

C-kit+ CPC from rat myocardium samples were obtained using the modified method described previously [6]. CPC were isolated from Wistar male rats (250-300 g). Animals were deeply narcotized by isoflurane inhalation, and the heart was excised, washed in sterile PBS, minced with scissors to 2-3 mm^3^ pieces, and incubated for 15 min in a mixture of 0.1% collagenase A (Roche Diagnostics, USA) and 0.2% trypsin (Invitrogen, USA). Minced heart pieces were cultured to establish cell outgrows cultures over 10 days using DMEM/F12 growth medium supplemented with 10% FBS, 10 ng/ml LIF, 100 U/ml each of penicillin and streptomycin, and 2 mM L-glutamine to generate explant culture. Every 3^rd^ day half volume was replenished by fresh explant medium. C-kit+ CPC were isolated from the cell outgrowth of the explants by immunomagnetic selection using a magnetic separator and the manufacturer's guidelines provided Milteniy Biotec. First, hematopoietic cells were depleted from outgrowth cells using CD45 antibodies (cat#554875, BD, USA) and magnetic immunobeads (cat#130-048-401, Milteniy Biotec, USA). The CD45 cells were then sorted for c-kit with a specific anti-c-kit antibodies (cat#sc-5535, Santa Cruz, USA) and magnetic immunobeads (cat#130-048-602, Milteniy Biotec, USA). Isolated cells were cultured on fibronectin-coated dishes in DMEM/F12 medium supplemented with 10% FBS, 100 U/ml each of penicillin and streptomycin, 2 mM L-glutamine, 2% B27 supplement, 1x insulin-transferrin selenium, and the following human growth factors: 20 ng/ml bFGF, 20 ng/ml EGF, and 10 ng/ml LIF.

### 2.3. Immunophenotype Analysis by FACS

To verify the purity and immunophenotype of CPC flow cytometry analysis was used. Isolated CPC were analyzed by FACS at early passage 2 cells: being detached, the cells were centrifuged (200*g*, 5 min), the supernatant was removed, and the pellet was resuspended and incubated for 30 min at +4°C in 1% BSA/PBS with antibodies against one of the following antigens: c-kit (cat#sc-5535, Santa Cruz, USA), CD31 (cat#sc-9095, BD, USA), CD34 (cat#sc-9095, Santa Cruz, USA), or CD45 (cat#554875, BD, USA). After incubation cells were centrifuged (200*g*, 5 min), twice washed with PBS, and resuspended in 1% BSA/PBS with secondary AlexaFluor488-conjugated antibodies (cat#A11001 and A11008, Invitrogen, USA). After a 30 min incubation at +4°C cells were washed, resuspended in PBS, and studied using FACS Canto II (BD, USA).

### 2.4. Immunophenotype Analysis by Immunocytochemistry

For* in vitro* staining, cells were plated on fibronectin-coated coverslips overnight, fixed with 4% paraformaldehyde, permeabilized with 0.1% Triton X-100 in PBS, and incubated with a rabbit polyclonal anti-c-kit antibody (cat #sc5535, Santa Cruz, USA, 1:100, 2h at 37°C), Oct3/4 (cat #ab18976, Abcam, USA, 1:100, 2h at 37°C), and Nanog (cat #sc33760, Santa Cruz, USA, 1:100, 2h at 37°C), followed by secondary anti-rabbit Alexa Fluor 488/594 antibodies (cat #A11001, A21207, all Invitrogen, USA, 1:800, 1h at 37°C); nuclei were counterstained by DAPI.

### 2.5. Differentiation Assay

Isolated c-kit+ CPC were expanded in a well-described way and seeded at passage 3 on culture slides (20000 cells/cm^2^) in *α*-MEM (Gibco, USA) supplemented with 10% FBS and 10 *μ*M of 5-azacytidine. Cells were incubated for 72 h with daily medium change and washed, and *α*-MEM/10% FBS with 10 ng/ml TGF-*β* was added for 21 days with medium change every 72 h. Differentiation was assayed by immunostaining for myosin heavy chains (cat #ab50967, Abcam, USA, 1:100, 1h at 37°C) and phalloidin-AlexaFluor488 (cat#12381, Invitrogen, USA, 1:100, 1h at 37°C) followed by secondary anti-mouse Alexa Fluor 488 antibodies (cat #A11001, Invitrogen, USA, 1:800, 1h at 37°C). To induce the endothelial differentiation c-kit + CPC were seeded at passage 3 on culture slides (20,000 cells / cm2) in DMEM-F12 medium containing 1% fetal bovine serum, 1% penicillin/streptomycin, 2 mM L-glutamine, 10^−8^ M dexamethasone, and 10 ng/ml growth factor VEGF. The cells were being cultured for 21 days. Differentiation medium was replaced every 48 hours. Endothelial differentiation was tested by immunostaining for PECAM (CD31) (cat#550300, BD, USA, 1:100, 1h at 37°C), followed by secondary anti-mouse AlexaFluor488 antibodies (cat #A21203, Invitrogen, USA, 1:800, 1h at 37°C). To induce smooth muscle differentiation c-kit + CPC were seeded at passage 3 on culture slides (20,000 cells / cm2) in DMEM-F12 medium containing 1% fetal bovine serum, 1% penicillin / streptomycin, 2 mM L-glutamine, 10^−8^ M Dexamethasone, and 40 ng/ml growth factor PDGF BB. The cells were being cultured for 21 days. Differentiation medium was replaced every 48 hours. Smooth muscle differentiation was tested by immunostaining for Gata 6 (cat#sc9055, Santa Cruz, USA, 1:100, 1h at 37°C) and smooth muscle actin FITC (сat#F3777, Sigma-Aldrich, USA, 1:100, 1h at 37°C), followed by secondary anti-rabbit AlexaFluor488 antibodies (cat #A11008, Invitrogen, USA, 1:800, 1h at 37°C).

### 2.6. Generation of Cell Sheets From c-Kit+ CPC

The cell sheets from passage 3 c-kit+ CPC were formed using thermoresponsive polyNIPAM-coated 12-well UpCell™ plates (cat#Z688835, Sigma-Aldrich, USA). Cultured CPC were labeled by CM DiI red (cat#C7000, ThermoFisher Scientific, USA) and then incubated on thermoresponsive dishes at 37°C for 72 hours. The CM DiI-labeled CPC spontaneously detached from the dish surface following incubation at 20°C for 30 minutes, yielding a CPC sheet.

### 2.7. Histologic Analysis of Cell Sheets

After detachment cell sheets were frozen in Tissue-Tek (Sakura Finetek, USA) and 7 *μ*m sections were obtained. Cross sections were formalin-fixed and stained by primary antibodies against Connexin43 (cat#71-0700, ThermoFisher Scientific, USA), Ki-67 (cat#ab16667, Abcam, USA), and Gata 4 (cat#PA5-29663, Thermo Fisher Scientific, USA) cleaved caspase-3 (cat#9664S, Cell signaling, USA) for 1 hour. To visualize ECM components cell sheet sections were incubated with a polyclonal antibodies specifically reactive with collagen I (cat#2150-1908, Bio-Rad, USA), collagen 3 (cat#2150-1948, Bio-Rad, USA), and the fibronectin (cat#6328-250, Abcam, USA). After washing the slides were stained for 40 min by the corresponding secondary antibodies (cat#A11001, A11008, A21203, or A21207, all Invitrogen, USA, 1:800, 1h at 37°C).

### 2.8. Animal Model of MI and Cell Sheet Transplantation

Myocardial infarction was induced in adult male Wistar rats in the way described previously [7]. Narcotized animals were provided with assisted ventilation and after heart isolation were subjected to permanent ligation of the anterior descending artery. The rats were randomly assigned to the following three groups: transplantation of cell sheet (*n* = 22), control (*n* = 28), or a sham operation (*n* = 20). Cell sheet was placed on the epicardium of the ischemic area and fixed by *«*Tisseel*»* fibrin sealant (Baxter, USA), then the heart was placed back to the chest cavity, muscles and skin were sutured, and the animals were ventilated until spontaneous breathing restored. Control animals underwent the same procedures without cell sheet transplantation. None of the animals died as a result of cell sheet grafting. The rats were taken care of for 14 or 60 days, when they were sacrificed in a humane manner.

### 2.9. Morphometric Analysis

Hearts were excised under deep anesthesia with inhalation of 5% isoflurane. Before the hearts were harvested, 0.1 ml saturated KCl had been injected into the left ventricular chamber to arrest them in diastole. The atriums and large vessels were resected and washed with normal saline, embedded in OCT compound, and frozen in liquid nitrogen. The hearts were stored at -70 C and sliced (7 *μ*m thickness with 300 *μ*m interval between sections) transversely from the apex to the base of left ventricular. All sections were stained using Mallory method. Mallory stain solutions A (1% acid fuchsin), B (1% phosphomolybdic acid), and C (2% orange G, 0.5% methyl blue, and 2% oxalic acid) were prepared on distilled water. Fixed and washed slides were incubated in solution A (2 min), solution B (4 min), and solution C (15 min). Slides were rinsed with distilled water between dyes, dehydrated, and mounted using a xylene-based medium. Whole sections were photographed and then used for morphometry analysis in NIH ImageJ freeware. Myocardial infarction measurement of length, including epicardial and endocardial infarction lengths, and epicardial and endocardial circumferences were performed manually using digital images and further measured automatically by the computer. To estimate the infarction lengths, endocardial infarct length was taken as a length of endocardial infarction scar surface that included >50% of the entire myocardial thickness and epicardial infarction length, as the length of the transmural infarction region. To obtain epicardial infarction ratio, the sum of epicardial infarction lengths from all sections was divided by the one of epicardial circumferences from all sections. Endocardial infarction ratio was calculated in a similar way. Infarction size derived from this approach was calculated in the following way: [(epicardial infarction ratio+endocardial infarction ratio)/2]×100 [8]. To measure the wall thickness, average thickness of three equal segments, which the infarction wall comprises, was taken into account. To quantitate both the degree of LV dilation and the degree of infarction wall thinning, the LV expansion index was calculated using a modified method of Hochman and Choo [9]: Expansion index = (LV cavity area/total area) x (noninfarcted region wall thickness/risk region wall thickness). To calculate the transmurality index, the method of Hochman and Choo [9] was used. The equation assessed the degree, to which the infarction extended completely through the left ventricular wall to touch the epicardium (Transmurality (%)=Infarction length touching epicardium/total infarction length).

### 2.10. Histologic Evaluation of Cell Sheet Vascularization, CPC Differentiation, and Migration

The frozen sections were fixed with formalin for 20 min and washed in PBS (5 min). After washing slides were blocked by 10% normal donkey serum (30 min). Antibodies were diluted in blocking solution (1% BSA in PBS) and sections were immunostained using antibodies against rat Ki-67 (cat#ab16667, Abcam, USA) for 1 h, then washed, and secondary stained with AlexaFluor488-conjugated antibodies (cat#A11001, Invitrogen, USA). To analyze endothelial differentiation cryosections were fixed and immunostained with antibodies against rat ETS1 (cat#ab186844, Abcam, USA), PECAM (CD31) (#550300, Becton Dickinson, USA), for cardiomyocyte differentiation, with antibodies against rat Nkx2.5 (cat#ab214296, Abcam, USA), MHC (#ab50967, Abcam, USA) for 1 h, washed, and then stained with secondary Alexa Fluor 488-conjugated antibodies (cat#A11001, Invitrogen, USA, 1:800, 1h at 37°C). All sections were counterstained with DAPI. The migration properties of the transplanted CM DiI+ CPC were examined with Zeiss Axiovert 200 M fluorescent microscope on the frozen sections, obtained at 7 and 14 days after cell sheet transplantation.

### 2.11. CD31 and *α*-SMA Immunostaining and Blood Vessel Density Evaluation

Sections were fixed in ice-cold acetone for 20 min, air-dried, and washed in PBS (5 min). After washing slides were blocked by 10% normal donkey serum (30 min). Antibodies were diluted in blocking solution (1% BSA in PBS) and sections were incubated with mouse anti-rat CD31 antibody (1:100, 1 hr). Then slides were washed in PBS (3×5 min) and incubated in a mixture of goat anti-mouse AlexaFluor594-conjugated antibodies (Cat#A-11005, Life technologies, USA, 1:800, 40 min) and anti-*α*-SMA FITC-conjugated antibodies (Cat#F3777, Sigma-Aldrich, USA, 1:50, 1 hr). At the end of incubation nuclei were stained with DAPI and sections were mounted under coverslips. Microphotographs were taken under 200x magnification in random fields of view located in peri-infarction areas of the section. Vessel counts were performed “blindfolded” by two independent persons manually. Capillary density analysis included CD31-positive structures per mm^2^; arteriolar counts per mm^2^ were estimated as number of *α*-SMA-positive vessels with clearly visible CD31-positive inner layer. Vessels with a CD31 marker having an internal lumen were also calculated for mm^2^.

### 2.12. Assessment of Cell Proliferation and Myofibroblasts Distribution in Myocardium Sections

Sections were fixed in formalin for 20 min and washed in PBS (5 min). After washing slides were blocked by 10% normal donkey serum (30 min). Antibodies were diluted in blocking solution (1% BSA in PBS) and sections were incubated with mouse anti-rat Ki-67 (cat#ab16667, Abcam, USA, 1:100, 1 hr). Then slides were washed in PBS (3×5 min) and incubated in a mixture of goat anti-mouse Alexa Fluor 594-conjugated antibodies (Cat#A11005, Life technologies, USA, 1:800, 40 min). At the end of incubation nuclei were stained with DAPI and sections were mounted under coverslips. Cardiomyocytes were visualized by staining with Troponin I antibodies (Cat#sc15368, Santa Cruz, USA; 1:400, 2h at 37°C), followed by donkey anti-rabbit Alexa Fluor 488 antibody (Cat#A21202, Invitrogen, USA; 1:800); nuclei were stained by DAPI. An additional method for assessment of cardiomyocytes proliferation was staining with primary anti-Ki-67 antibody (cat # 14-5698-82, ThermoFisher Scientific, USA) and anti-laminin antibodies (cat # ab11575, Abcam, USA). Then slides were washed in PBS (3–5 min) and incubated in a mixture secondary AlexaFluor594/AlexaFluor488-conjugated antibodies (Cat# R37117 and A-11006, Life technologies, USA, 1:800, 40 min). At the end of incubation nuclei were stained with DAPI and sections were mounted under coverslips. Data was expressed as the density of Ki67+ proliferated cells per mm^2^ of border and infarct areas. The proliferated cardiomyocytes number was calculated as a percent of Ki67+ cardiomyocytes to the total number of cardiomyocytes in the damage zone.

Myofibroblasts distribution in infarcted left ventricular wall was analyzed by staining with *α*-smooth muscle actin FITC-conjugated antibody (Cat# F3777, Sigma, USA; 1:100, 1h at 37°C) and vimentin antibody (Cat# V6630, Sigma, USA; 1:100, 1h at 37°C), followed by secondary anti-mouse AlexaFluor594 antibodies (cat #A21203, Invitrogen, USA, 1:800, 1h at 37°C); nuclei were stained by DAPI. Data was expressed as number of myofibroblasts per mm^2^ of damage zone surface in section.

### 2.13. Fluorescent In Situ Hybridization (FISH) Analysis

To analyze the fate of the transplanted male CPC, single-labeled Y-chromosome was detected using fluorescent* in situ* hybridization (FISH) according to the manufacturer's protocol. The frozen heart was cut in 7-*μ*m sections. After treatment with pepsin and air-drying, denatured rat Y-chromosome probe was added to each section and covered with coverslip. After overnight hybridization at RT°C, the coverslip was removed and serial washings were performed. Male and female rat heart tissue sections were used as positive and negative controls, respectively, in the FISH staining procedure. The fate of transplanted CPC in the heart was assessed by double staining with a green fluorochrome-labeled Y-chromosome probe and a specific monoclonal antibody against the cardiac-specific marker typical of *β*-myosin heavy chains (#ab50967, Abcam, USA) and PECAM (CD31) (#550300, Becton Dickinson, USA). Nuclei were counterstained with DAPI.

### 2.14. Microscopy and Image Analysis

Sections were analyzed using a Zeiss Axiovert 200 M fluorescent microscope and Axiovision 3.1 software (both, Carl Zeiss, Germany) for raw image processing and conversion.

### 2.15. Statistical Analysis of Data

Data are expressed as mean±SD or SEM. Statistical significance of the difference was estimated by Mann–Whitney* U*-test in Statistica 8.0 (Statsoft, USA).

## 3. Results

### 3.1. Characteristics of CPC

**Figure 1:**
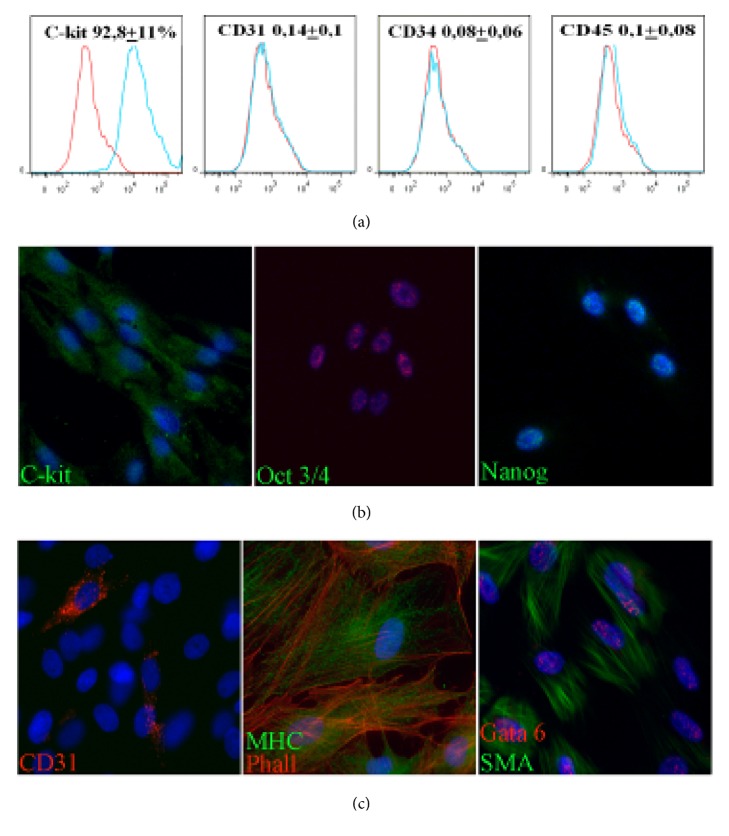
Isolation and characterization of rat cardiac progenitor cells (CPC). (a) Flow cytometry analysis of c-kit and hematopoietic markers (CD34, CD45 in CPC after isolation). Control IgG staining profiles are shown red border line histograms, while specific antibody staining profiles are shown blue in histograms. Data is expressed as mean+SD. (b) Expression of c-kit and pluripotency markers (Oct-3/4, Nanog) in CPC culture visualized by immunocytochemistry. (c) In vitro differentiation potential of isolated CPC toward cardiovascular images. Representative images of immunofluorescent staining in induced CPC for markers of endothelial (CD31 (PECAM)), cardiomyocytes (myosin heavy chains), and smooth muscle cells (Gata 6, smooth muscle actin).

C-kit+ CPC were obtained from rat myocardium using explant culture followed by immunomagnetic selection using antibodies CD117/c-kit as a CPC marker. Flow cytometry analysis has confirmed that c-kit was present on the surface of more than 90% of isolated CPC (Figure 1(a)). Furthermore, cells in culture lacked CD45 suggesting that isolated cells did not express markers of bone marrow origin. Obtained CPC population contained neither PECAM (CD31) nor CD34, which provided excluded presence of endothelial progenitors or mature endothelial cells in obtained culture [10–12]. Immunofluorescent analysis showed sporadic cells expressing stem markers Oct-4 and Nanog (Figure 1(b) known to be related to cell's ability for self-renewal and its early differentiation status in culture.

We next investigated differentiation capacity toward cardiovascular lineages. By immunocytochemistry we confirmed ability of c-kit+ CPC to express markers of endothelial (PECAM), smooth muscle (smooth muscle actin), and cardiomyocytes (myosin heavy chain) after induction by specific culture media for 2 weeks (Figure 1(c)). Our data fully corresponded with the previously described characteristic of mammalian CPC [4, 13, 14].

### 3.2. Characteristics of the Cell Sheets

**Figure 2:**
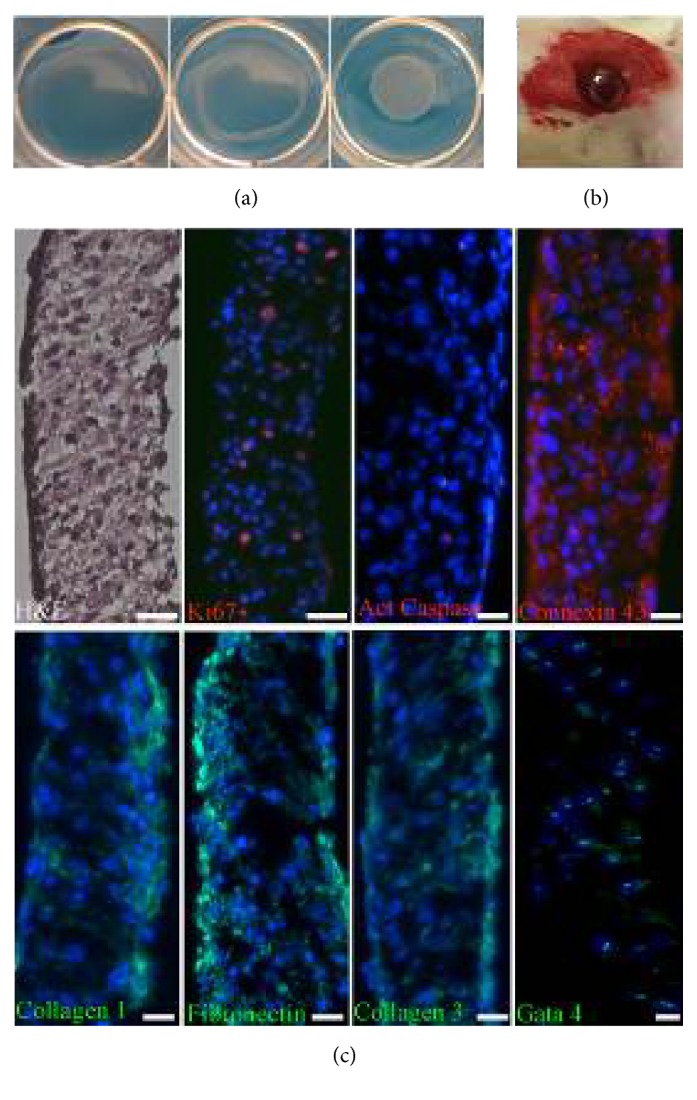
Isolation and characterization of cell sheets from c-kit+ CPC. (a) CPC sheets formed after 3 days of culture detached at room temperature onto thermoresponsive surfaces yielding a scaffold-free monolayered cell sheet. (b) Hematoxylin–eosin staining shows that the cell sheet represents a well-organized structure. CPC in the cell sheets survived, retained the ability to proliferate (Ki67), expressed progenitor cell marker Gata 4, interconnected with each other via connexin-43 gap junctions, and were surrounded extracellular matrix proteins (collagen 1, collagen 3, and fibronectin). (c) Macroscopic visualization of epicardially attached CPC sheet after implantation.

Cell sheets were obtained by culturing of CPC on plates with UpCell™ surface coated by thermosensitive polymer. At 37°С dish surface containing PIPAAm becomes hydrophobic promoting cell adhesion and growth and at temperature under than 32°С turns hydrophilic and polymer absorbs water and expands resulting in detachment of cell sheets (Figure 2(a)). Cell sheet diameter after detachment was 0.98±0.36 cm^2^ and histological analysis of obtained cell sheets using has shown that constructs consist of several cell layers with an average thickness of 111.8 ± 24 *μ*m. We also detected abundant expression of connexin-43 and formation of gap junctions (Figure 2(b)). Certain amount of cells within construct expressed Ki–67 proliferation marker (73.9 ± 47 per 1000 cells) which taken along with absence of apoptosis marker (activated caspase 3) indicated effective support of cell proliferation and viability in cell sheets. About 17±9% of CPC expressed transcriptional factor Gata 4 which characterized early stages of cardiac or vascular differentiation. Structure of cell sheets contained extracellular matrix proteins (fibronectin, type 1 and type 3 collagens) produced by cells in the course of cell sheet formation (Figure 2(b)).

### 3.3. Integration and Survival of CPC after Transplantation

**Figure 3:**
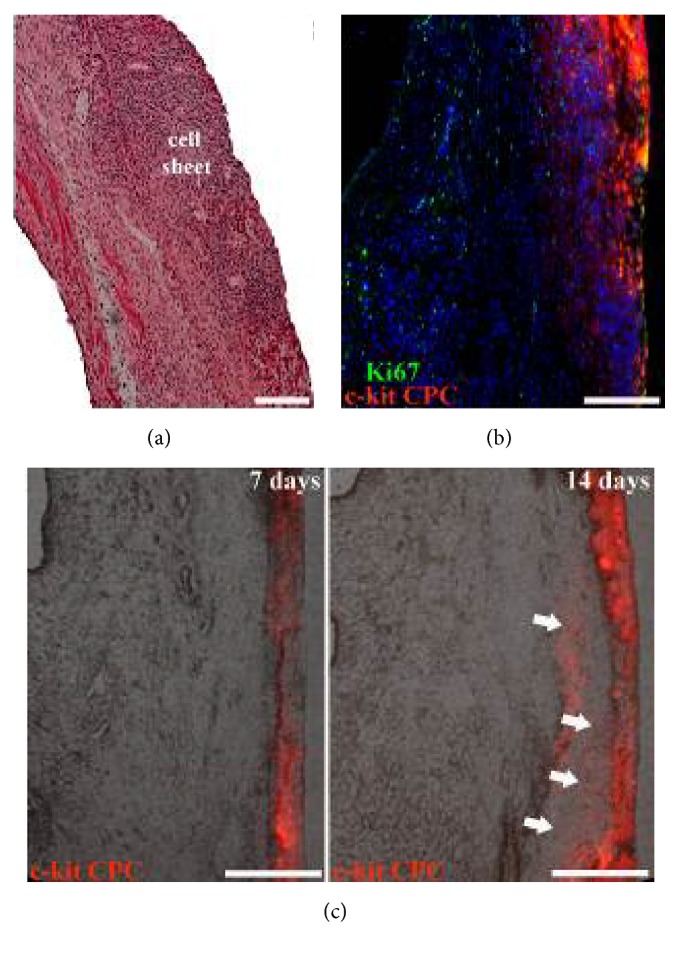
Characterization of CPC cell sheet after epicardial delivery. (a) Hematoxylin–eosin staining shows stable attachment and engraftment of cell sheet on the epicardial surface of the heart. (b) CM-DIL+ CPC retained ability to proliferate after transplantation. (c) Numerous CM-DIL+ CPC were detected within the epicardial graft and were able to migrate to underlying myocardium.

Posttransplantation survival of stem/progenitor cells is considered to be a major factor determining efficacy of cell therapy. We assessed cell fate after transplantation using vital membrane dye CM-DIL (Figure 3). Fourteen days after transplantation we detected stained CPC in all sections with integrated cell sheet. We found pronounced migration of CPC from the cell sheet into damaged myocardium between 7 and 14 days. On average, by the fourteenth day the area of CPC spread was 3-fold larger than that of the initial cell sheet (Figure 3(c)). Furthermore, transplanted CPC appeared to preserve their ability for proliferation (Ki67 marker) (Figure 3(b)). Mean number of proliferating cells was 548.9 ± 103 per mm^2^ of the cell sheet area. The implant included sporadic labeled cells positive for activate caspase-3 suggesting a very low level of apoptosis in the graft.

### 3.4. Size of Infarction and Cardiac Remodeling Parameters

**Figure 4:**
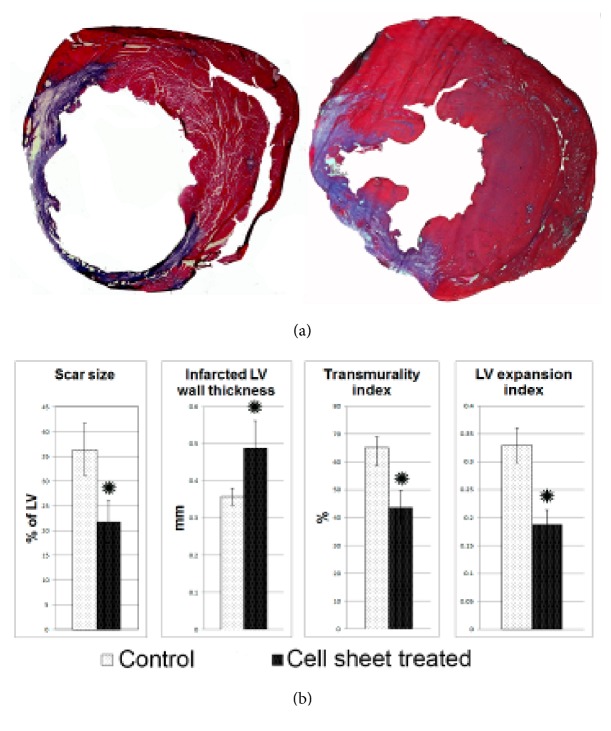
Morphometric evaluation of the ventricular in rats 14 days after myocardial infarction and cell sheet therapy. (a) Representative images of Mallory-stained cross sections of myocardium. Collagen fibers are stained blue and viable tissue stained purple. (b) Histograms summarizing quantitative data: infarct size, infarct wall thickness, transmurality and left ventricular expansion indices 14 days after infarction. Data is presented as mean±SD. *∗*p<0.05 versus control.

We next assessed effects of cell sheet epicardial implantation on postinfarction cardiac remodeling. We have shown that size of the postinfarction scar reduced approximately 2-fold compared to control group (Figure 4). On day 14 after surgery left ventricle (LV) wall thickness appeared to be approximately 13% higher in CPC sheet group. We also found a significant decrease in severity of transmural damage. Thickening of LV anterior wall in CPC sheet group wall coincided with reduction of LV dilation. Indeed, after CPC sheet transplantation LV dilation index was significantly lower compared to control which may be considered as a major factor contributing to reduction of postinfarction LV remodeling and alleviation of cardiac failure after CPC sheet implantation.

### 3.5. Analysis of Cell Fate after CPC-Based Cell Sheet Delivery

**Figure 5:**
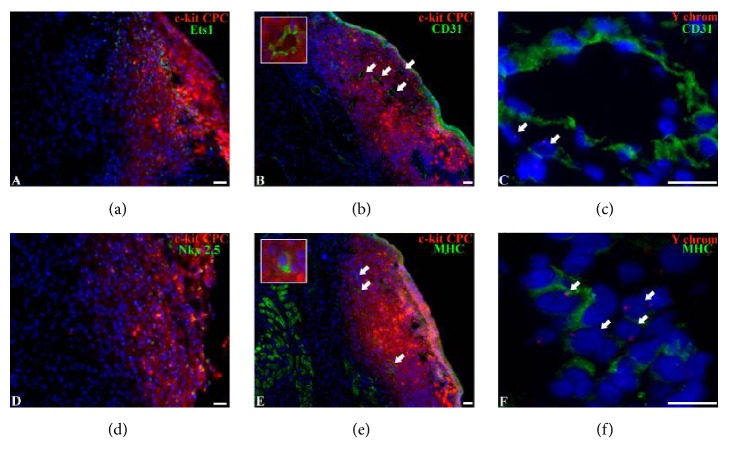
Differentiation and Y-chromosome detection in CPC-based cell sheets. (a, b) Differentiation of CPC in cell sheets toward vascular lineage. Representative images of immunofluorescence staining of the heart sections with antibodies against endothelial marker Ets-1 (a) and CD31 (b). Grafted cells were identified by CM-DIL (red fluorescence). Arrows indicate CM-DIL+ vessels. (c) Y-chromosome detection in CPC-derived endothelial cell. Representative images of immunofluorescent staining of heart sections with antibodies against endothelial marker CD31 and Y-chromosome. Arrows indicate Y-chromosome+ endothelial cells. (d, e) Differentiation of CPC in cell sheets toward cardiomyocyte lineage. Representative images of immunofluorescent staining of heart sections with antibodies against cardiomyocyte markers Nkx2.5 (d) and MHC (e) (*β*-myosin heavy chain). Grafted cells were identified by CM-DIL (red fluorescence). Arrows indicate CM-DIL+ cardiomyocytes. (f) Y-chromosome detection in cardiomyocytes. Representative images of immunofluorescence staining of the heart sections with antibodies against cardiomyocyte marker MHC and Y-chromosome probe. Arrows indicate Y-chromosome+ cardiomyocytes.

We found that after transplantation to ischemic myocardium CPC were capable of undergoing multilineage differentiation and replace vascular cells and cardiomyocytes (Figure 5). By the fourteenth day after transplantation cell sheet cells integrated to the damage myocardium and formed a vascularized tissue that contained numerous arterial and venous vessels of different diameter. In cell sheers we detected colocalization of CM-DIL with endothelial transcription factor ETS1 (Figure 5(a)) and mature endothelial cells marker CD31 (Figure 5(b)) suggesting* de novo* vessels formation by CPC. We also found clusters of labeled CPC expressing cardiomyocyte differentiation markers Nkx 2.5 (Figure 5(d)) and MHC (*β*-myosin heavy chains) (Figure 5(e)), suggesting* in situ *differentiation of transplanted CPC. Sex mismatch analysis was performed in order to confirm presumable differentiation of CPC grafted on the infarcted part of myocardium. Cell sheet consisting of male rat CPC were transplanted into female rat hearts with subsequent identification of Y-chromosome in the cells expressing endothelial or cardiomyocyte differentiation markers (Figures 5(c) and 5(f)). Y-chromosome was detected in CD31+ cells, which form endothelial lining of blood vessels. Only occasional cardiomyocytes were detected with Y-chromosome indicating limited formation of cardiomyocytes from delivered CPC.

### 3.6. Estimation of Transplanted CPC Sheets Influence on Angiogenesis in Myocardium

**Figure 6:**
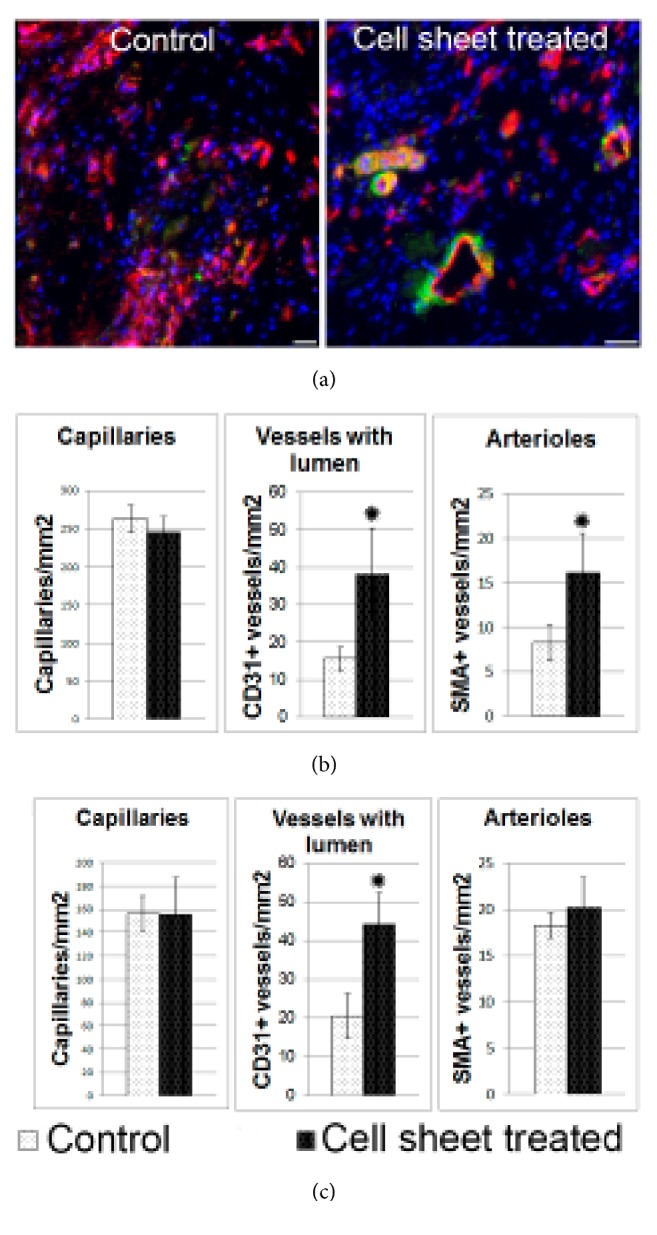
Angiogenesis in left ventricle 14 days after myocardial infarction and CPC sheet delivery. (a) Representative images of capillaries and arterioles in border zone in control group and after cell sheet treatment. Two weeks after epicardial delivery of CPC sheets sections were costained with antibodies against endothelial marker CD31 (red) and smooth muscle marker-smooth muscle actin (green); nuclei are counterstained by DAPI (blue). (b) Bar graphs represent analysis of density for capillaries, vessels with lumen and arterioles in border zone. (c) Bar graphs graphs represent analysis of density for capillaries, vessels with lumen and arterioles in infarct zone. Data is presented as mean±SD. *∗*p<0.05 versus control.

Vessel density was assessed by staining of myocardium sections for endothelial cells (CD31) and smooth muscle cells (smooth muscle actin) (Figure 6(a)). We found significant differences in vessels counts between control and cell sheet treated groups. In animals treated by CPC sheet transplantation we found increased number of CD31+ vessels with lumen located in border and infarct zones (Figures 6(b) and 6(c)). Arteriolar density (CD31+/SMA+ vessels) tended to be increased in cell sheet group, but difference was statistically significant only in the border zone. However, we did not find significant difference of capillary counts in experimental groups.

### 3.7. Estimation of Cell Proliferation in Scar Zone after the Transplantation of CPC

**Figure 7:**
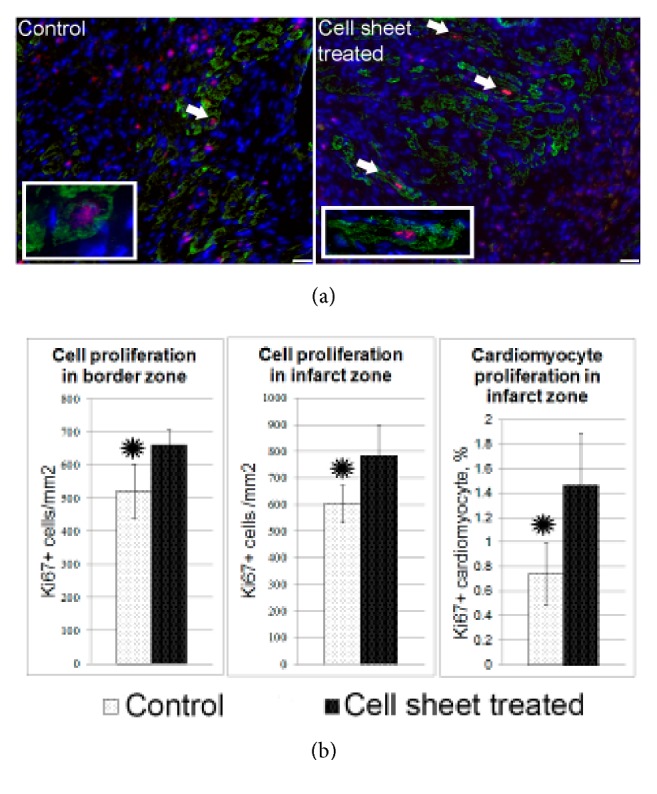
Total and cardiomyocyte proliferation in the left ventricle wall 14 days after myocardial infarction and delivery of CPC-based cell sheets. (a) Representative images of cardiomyocyte proliferation in control group and after cell sheet treatment. Two weeks after epicardial delivery of the CPC sheet, the heart sections were costained with the antibodies against the cell-proliferation-associated antigen-Ki67 (red fluorescence) and cardiomyocyte marker, Troponin I (green fluorescence). Combined red and green fluorescence and DAPI-stained nuclei (blue) are shown in merged images. Arrows indicate costained proliferated cardiomyocytes. (b) Bar graphs show quantitative data of the number of total proliferating cells in border or infarct zones and cardiomyocyte proliferation. Data is presented as mean±SD. *∗*p<0.05 versus control.

Proliferative stage accompanies ischemic damage and occurs at late stages after necrosis. It provides reparative regeneration in heart proliferation of connective tissue components and specific cell reaches its peak after the damaged zone is debrided from necrotic cells by tissue macrophages. Histological analysis has shown that cell sheet transplantation activates cell proliferation in scar and border zones (Figure 7(b)).

We found islets of preserved myocardium with occasional proliferating Ki67+ cardiomyocytes (Figure 7(a), Figure S1) under grafted cell sheet in scar zone of left ventricle. As compared to total cardiomyocyte count percentage of proliferating cardiomyocytes was significantly higher after cell sheet transplantation than in the control group. However, proliferating cardiomyocytes did not contain fluorescent dye thus excluding transplanted CPC as origin of these Ki67+ cardiomyocytes. Enhanced proliferative activity of cardiomyocytes could be attributed to influence of paracrine factors produced by implanted CPC.

## 4. Discussion

Stem cell transplantation is one of the most promising treatments for myocardial lesions including CHF. However, effects of stem cell administration on cardiac function in the clinical setting have not met expectations. Poor survival of transplanted cells due to cell injury during isolation procedure, apoptosis, exposure to ischemic conditions, inflammation, and immunological rejection is the major limitation of therapeutic efficacy after cell transplantation [15–21]. Mouse model of MI demonstrated that death of transplanted cells markedly impairs cardiac function, while boosting survival of cells improves it due to apoptosis suppression [22, 23]. Therefore, increasing survival of transplanted cell is paramount to improve therapeutic potential of cardiovascular cell therapy. Several studies demonstrated that transplantation of cell sheets improved survival compared to dispersed cell injection [5].

In this study, we evaluated the efficacy of epicardial implantation of c-kit+ CPC sheets in a rat model of MI. Although last studies have found limited evidence that these cells differentiate to mature functional cardiomyocytes, most preclinical and clinical studies suggest that infusion or injection of c-kit+ CPC confers therapeutic benefits to the injured heart [24]. Mechanisms of these benefits remain obscure but may involve paracrine actions, including exosome-derived effects on damage cardiac tissue [25–27]. Furthermore, some authors consider that c-kit+ cells found in the adult heart exhibited epicardium-derived, noncardiomyogenic precursors with a mesenchymal phenotype which are capable of contributing significantly to smooth muscle cells and endothelial cells, but not to cardiomyocytes.

Taking that into account we hypothesized that epicardial implantation of c-kit+ cardiac progenitor cell sheets would enhance cell survival, engraftment, and structural recovery of infarcted myocardium by stimulating endogenous mechanisms of regeneration by paracrine activity and by participation of transplanted cells in myocardial vascularization. Besides that, we believe that epicardial implantation of cell sheets would promote contribution of endogenous epicardial cells to cardiac repair.

In the present study, we obtained c-kit-positive CРCs from rat myocardium using culturing myocardial tissue ‘as explants' followed by immunomagnetic selection. Obtained c-kit-positive cells had no hematopoietic markers, were able to preserve the phenotype of undifferentiated cells under specific culture conditions, express stem cell markers (Figure 1(a)), were clonogenic, and were able to undergo induced differentiation in endothelial, cardiomyocyte, and smooth muscle cells lineages (Figure 1(c)). However, we failed to obtain fully differentiated cardiomyocytes and only several cells expressing markers of cardiogenic lineage were found in c-kit+ cells culture after induction of cardiomyocyte differentiation. On the contrary, the cells with the phenotype of mature endothelial cells could be obtained, showing that c-kit-positive CРCs are more prone to endothelial than to cardiomyocyte differentiation (Figures 1 and 5)

We have observed that CРC included to the cell sheets, which were obtained using thermosensitive dishes, proliferated well, did not enter apoptosis, formed cell-to-cell contacts through connexin 43, express differentiation markers (GATA4), and secreted extracellular matrix proteins, i.e., type 1 and 3 collagens and fibronectin (Figure 2(b)).

Epicardial delivery of c-kit+ CPC as a scaffold-free cell sheet facilitates cell survival after transplantation onto the infarcted heart. CРC sheets engrafted successfully into the epicardium over ischemic area. A significant percentage of implanted CPC actively proliferated and migrated from the cell sheet to the site of infarction that could probably be caused by increasing production of growth factors and cytokines in a damaged ischemic myocardium (SDF-1, HGF, VEGF, and IGF-1). As it was shown previously SDF-1-CXCR4 axis plays, at least in part, a role in cardiac progenitor cell migration from cell sheet to the infarcted myocardium [28].

Analyzing the secretion profile of cultured c-kit+ CPC, isolated from the right atrial appendage, resected during an elective coronary artery bypass graft, we found high level of secretion of angiogenic factors: HGF, VEGF, angiopoietin-1, SDF-1, IGF–1, uPA, and suPAR (unpublished data).

We have observed that the most of transplanted cells express the marker of vascular lineages, ETS-1 soon after transplantation suggesting their vasculogenic potential, and the implant area had been highly vascularized 2 weeks after transplantation (Figures 5(a) and 5(b)). Moreover, about 70-80% of blood vessels located in this area had been formed by CM-Dil labeled cells indicating possible differentiation of transplanted cells to vascular lineage. Thus, the induction of cell sheet vascularization soon after transplantation probably is the main mechanism of transplanted cell survival. It could be due to both the differentiation of implant cells to vascular cells and paracrine activity of these cells. These results are in full accordance with the data on the induced endothelial and SMC differentiation of c-kit+ CРC* in vitro*. Moreover, we have shown previously that c-kit+ CPC implanted in Matrigel to mice subcutaneously induced stronger implant vascularization than the same quantity of adipose-derived stem cells well known for their strong angiogenic properties (unpublished data) demonstrating pronounced angiogenic activity of c-kit+ CPC.

Although part of transplanted c-kit+ CРCs expressed the early markers of cardiomyocyte differentiation (Nkx2.5), the markers of more mature cardiomyocytes (MHC) were detected only in single cases. Sex mismatch study has also provided evidence of possible cardiomyocyte differentiation in a small proportion of transplanted c-kit+ CPC suggesting absence of significant cardiomyogenesis in c-kit+ CРCs implants.

Here we report that implanting CPC sheet onto infarcted myocardium promoted the decrease in the scar size and prevented cardiac wall thinning and LV remodeling at 2 and 8 weeks after transplantation (Figure 4, Figure S2). Moreover, c-kit+ CPC sheets transplantation significantly increases the survival rate of the animals within an 8-week observation period (Figure S3). This effect could be determined largely by a suppressed remodeling of the left ventricle, which could be due to a mechanical support provided by implanted cell sheet to the infarcted left ventricular wall. This supporting effect of cell sheet probably is due to a large number of myofibroblasts within the sheet detected at 8 weeks (Figure S4). Apart from the mechanical support, secretion of growth factors and exosomes by the implanted cells could be important. We observed significant myocardial neovascularization in border and infarct areas under implant. This induction of neovascularization may prevent ischemia-induced cell death and it has been shown that in case of ischemic cardiomyopathy improved myocardial perfusion is associated with reduced negative remodeling of the left ventricle [29]. Thus, CPC sheet implantation may prevent negative myocardial remodeling in part by stimulating the neovascularization of the infarction and the peri-infarction zone through paracrine mechanisms. Besides that, an increase in the total number of proliferating cells and in the number of proliferating cardiomyocytes in border zone, observed in our study, can also be determined by paracrine function of the implanted cells. Cardiomyocyte proliferation together with suppression of cardiomyocyte apoptosis may contribute to the increase in the area of viable myocardium in the damage zone thus in their turn preventing LV remodeling. It has been demonstrated that coculture of c-kit+ CPC expressing Gata 4 and mature rat cardiomyocytes favorably increases survival of cardiomyocytes* in vitro* and stimulates their contractive activity [30]. These effects are triggered by IGF–1, secreted by с–kit+Gata 4+ CPC, which activates signal cascade of IGF–1R/Akt, and suppressed cardiomyocyte apoptosis. HGF [31] and VEGF [32, 33], both secreted by c-kit+ CPC, also may induce cardiomyocyte survival.

Study limitation of our experiment in a rat model includes that a potential “inert control” might be included in future design using dermal fibroblasts or other “nonregenerative” cell type. While planning we encountered limited possibility of choosing “biologically inert cells” for transplantation. We considered skin fibroblasts, yet recent data shows that they compose an extremely heterogeneous population [34], where each subclone has unique paracrine and differentiation characteristics [35], which does not allow them to be considered as a correct comparison group with “close to nil” regenerative capacity. Moreover, fibroblasts have paracrine activity and may trigger events in damaged myocardium as well as produce ECM proteins that influence fibrosis and its span in most tissues.

Among possible mechanisms triggering cardiomyocyte proliferation in peri-infarct zone the release of exosomes by implant cells could not to be excluded. Regenerative effects of exosomes isolated from cardiac progenitor cells were demonstrated recently [36]. These effects were related to specific microRNA transferred by exosomes to cardiomyocytes.

Regenerative activity of CPC sheets can also be associated with paracrine activation of epicardial derived cells. In the adult heart, injury activates the epicardium that induces an embryonic-like response which includes epithelial to mesenchymal transition thus providing epicardial derived cells that migrate into the myocardium and participate in myocardial vascularization by differentiation into vascular cells as well as paracrine mechanisms [37].

Distorted microenvironment including changes in extracellular matrix (ECM) composition is also considered among factors inhibiting regenerative activity of cells grafted into myocardium by injection [38]. ECM is a critical component of microenvironment for cells and modulates their proliferation, viability, and differentiation. Scar formation is accompanied by changes in ECM composition: a sharp increase in amount of type I collagen and a dramatic decrease in amount of fibronectin and laminin. It is known that fibronectin is a key regulatory factor in CPC proliferation through the *β*_1_-Integrin-FAK-Stat3-Pim1 signal pathway [39]. Since integrin-mediated signaling influences several cellular signal pathways regulating differentiation, apoptosis, and proliferation [40], it can be suggested that deficiency of fibronectin in the infarction zone may produce negative effects on engraftment and proliferation of transplanted cells as well as endogenous progenitor cells. Therefore CPC sheet producing an optimal ratio of type 1 and type 3 collagen and fibronectin can contribute to endogenous regeneration process.

Thus, it may be suggested that prolonged secretion of growth factors and cytokines as well as exosomes release and ECM production by implanted CPC could render pleiotropic effects on various links of myocardial reparation/regeneration. Although numerous experiments showed increased LV ejection fraction (LVEF) after epicardial implantation of cells sheets consisting of various cell types [41, 42] no significant improvement of LVEF was observed during our experiments. Recently, use of LVEF as the first end point to evaluate the cell therapy effectiveness has been challenged as questionable, since this parameter may vary considerably depending on LV pre- and afterloads [43]. Presumably, stimulation of neovascularization and reduced LV remodeling could improve the survival rate to some extent, as compared to LVEF modification.

## 5. Conclusion 

Epicardial transplantation of cell sheets from c-kit+ CPC onto infarcted heart in rats considerably increased animal survival rate within 8-week follow-up period most probably due to reduction of left ventricle remodeling related to stimulation of angiogenesis and fibrosis decrease. Majority of CPC delivered using scaffold-free cell sheet survived and even proliferated after transplantation. Although no significant evidence for differentiation of implanted CPC to cardiomyocytes was found, we observed certain span of cardiogenesis within infarcted area. Described benefits are believed to be mediated by paracrine mechanism and further investigation is required to determine precise effector molecules by which CPC may increase survival rate of animals after MI. These results suggest that transplantation of c-kit+ CPC sheets as scaffold-free constructs is a promising tool for cell-based therapy of heart diseases.

## Data Availability

The data used to support the findings of this study are available from the corresponding author upon request.

## Supplementary Materials

Supplementary materials Supplementary materials contain additional figures, showing cardiac remodeling, animal survival, myofibroblasts activation, and cardiomyocyte proliferation after cell sheet transplantation.
