# Supplementary material for: C-Kit Cardiac Progenitor Cell Based Cell Sheet Improves Vascularization and Attenuates Cardiac Remodeling following Myocardial Infarction in Rats
Source: Biomed Res Int. 2018 Jun 25;2018:3536854. doi: 10.1155/2018/3536854 (PMC6036839; doi:10.1155/2018/3536854)
Supplement: Supplementary materials — contain additional figures, showing cardiac remodeling, animal survival, myofibroblasts activation, and cardiomyocyte proliferation after cell sheet transplantation. [file 3536854.f1.docx]

*
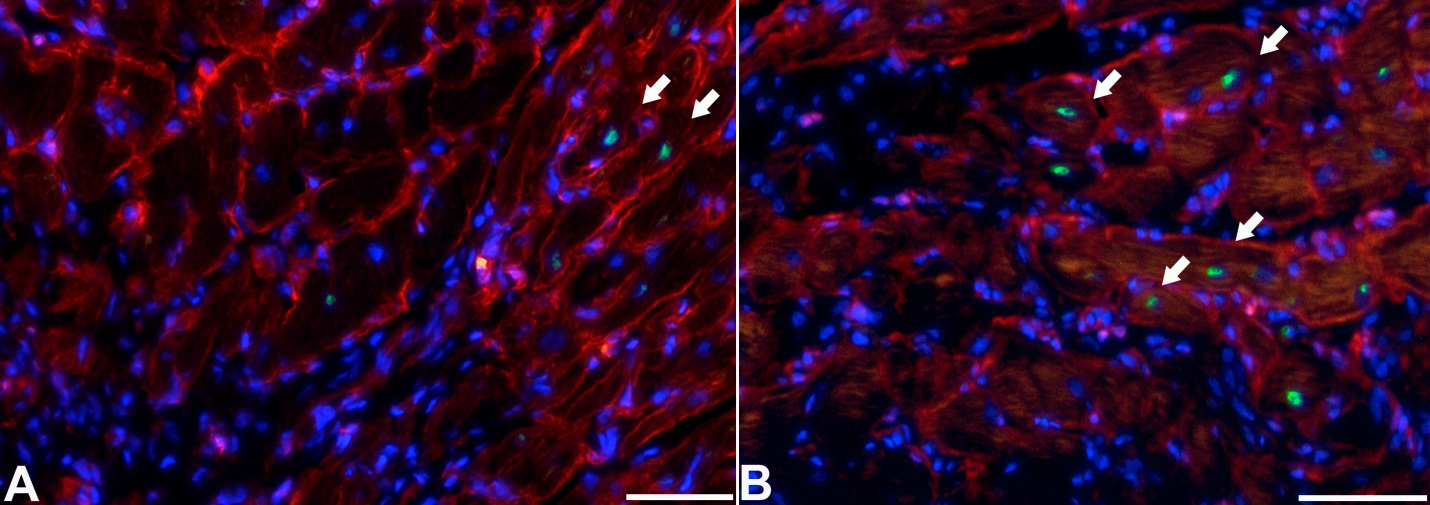
*

*Fig. S1. Cardiomyocyte proliferation in the left ventricle wall 14 days post myocardial infarction and delivery of CPC-based cell sheets.*

*Representative images of cardiomyocyte proliferation in control group (A) and after cell sheet treatment (B). Two weeks after epicardial delivery of the CPC sheet, the heart sections were co-stained with the antibodies against the cell-proliferation-associated antigen-Ki67 (green fluorescence) and laminin (red fluorescence). Combined red and green fluorescence and DAPI-stained nuclei (blue) are shown in merged images. Arrows indicate co-stained proliferated cardiomyocyte. Scale bar represents 100 μm.*
